# Improving compliance with guidelines may lead to favorable clinical outcomes for patients with non‐muscle‐invasive bladder cancer: A retrospective multicenter study

**DOI:** 10.1111/iju.15294

**Published:** 2023-09-04

**Authors:** Takuma Sato, Takeshi Sano, Sadafumi Kawamura, Yoshihiro Ikeda, Kazuhiko Orikasa, Takaki Tanaka, Atsushi Kyan, Shozo Ota, Satoru Tokuyama, Hideo Saito, Koji Mitsuzuka, Shinichi Yamashita, Yoichi Arai, Takashi Kobayashi, Akihiro Ito

**Affiliations:** ^1^ Department of Urology Tohoku University Graduate School of Medicine Sendai Miyagi Japan; ^2^ Tohoku Urological Evidence‐Based Medicine Study Group Sendai Japan; ^3^ Department of Urology Kyoto University Graduate School of Medicine Kyoto Japan; ^4^ Department of Urology Miyagi Cancer Center Natori Miyagi Japan; ^5^ Department of Urology Osaki Citizen Hospital Ōsaki Miyagi Japan; ^6^ Department of Urology Kesennuma City Hospital Kesennuma Miyagi Japan; ^7^ Department of Urology Hachinohe City Hospital Aomori Japan; ^8^ Department of Urology Shirakawa Kosei General Hospital Fukushima Japan; ^9^ Department of Urology Sendai Red Cross Hospital Sendai Miyagi Japan; ^10^ Department of Urology Iwaki City Medical Center Fukushima Japan; ^11^ Department of Urology National Hospital Organization Sendai Medical Center Miyagi Japan

**Keywords:** clinical outcome, guideline adherence, intravesical bacillus Calmette–Guérin, non‐muscle‐invasive bladder cancer, second transurethral resection

## Abstract

**Objectives:**

Clinical guidelines recommend that patients with non‐muscle‐invasive bladder cancer (NMIBC) should be treated with appropriate adjuvant therapy. However, compliance with guideline recommendations is insufficient, and this may lead to unfavorable outcomes. We aimed to investigate the level of adherence to guideline recommendations in patients with NMIBC and evaluate the outcomes of those who did and did not receive guideline‐recommended therapies.

**Methods:**

We performed a retrospective analysis of patients with histologically diagnosed NMIBC. The percentage of patients with intermediate‐ and high‐risk tumors who received adjuvant intravesical therapy or second transurethral resection (TUR) was calculated. Recurrence‐free survival was assessed in patients who did and did not receive the therapies. We conducted a propensity score‐matched analysis to compare outcomes between patients with intermediate‐risk and T1 NMIBC who did and did not undergo guideline‐recommended therapies.

**Results:**

Overall, 1204 patients from the Tohoku Urological Evidence‐Based Medicine Study Group and Kyoto University Hospital were included. Of patients with intermediate‐ and high‐risk tumors, 91.0% and 74.0% did not receive maintenance bacillus Calmette–Guérin (BCG), respectively. In both groups, significantly better recurrence‐free survival was found for patients treated with maintenance BCG. Among patients with T1 NMIBC, only 16.7% underwent guideline‐recommended therapies, that is, a second TUR and maintenance BCG. Significantly greater recurrence‐free survival was observed in patients who received guideline‐recommended therapies compared with propensity‐matched patients who did not.

**Conclusions:**

Guideline‐recommended therapies may contribute to improvements in outcomes for patients with NMIBC, suggesting that improvements in adherence to clinical guidelines may lead to favorable outcomes.

**Abbreviations & Acronyms**
BCG: bacillus Calmette–Guérin
CIS: carcinoma in situ
EAU: European Association of Urology
EORTC: European Organization for Research and Treatment of Cancer
IPIC: immediate postoperative instillation of chemotherapy
IQR: interquartile range
JUA: Japanese Urological Association
MIBC: muscle‐invasive bladder cancer
NMIBC: non‐muscle‐invasive bladder cancer
RCT: randomized controlled trial
RFS: recurrence‐free survival
TUR: transurethral resection
TURBT: transurethral resection of bladder tumor

## INTRODUCTION

Non‐muscle‐invasive bladder cancer (NMIBC) accounts for approximately 75% of newly diagnosed bladder cancer cases and has a 5‐year recurrence rate of 30%–80%.^1^ Tumor recurrence has a negative impact on health‐related quality of life and imposes heavy economic burdens on medical care expenditure; additionally, progression to muscle‐invasive bladder cancer (MIBC) is associated with death resulting from bladder cancer.^2^, ^3^

To reduce the risk of disease recurrence, various treatments, including intravesical bacillus Calmette–Guérin (BCG), immediate postoperative instillation of chemotherapy (IPIC), additional adjuvant intravesical chemotherapy, and second transurethral resection (TUR), have been performed after initial transurethral resection of bladder tumor (TURBT).^4^ Many large‐scale and high‐quality studies have been performed to assess the treatment efficacy of each therapy. The results of multiple randomized controlled trials (RCTs) revealed that maintenance BCG reduced the probability of disease recurrence and might contribute to decreasing the risk of progression.^4^, ^5^ In a recent systematic review and individual patient data meta‐analysis, IPIC reduced the 5‐year recurrence rate by 14%.^6^ A systematic review showed that the second TUR facilitated the detection of residual tumors after the initial TURBT and had the potential to prevent disease recurrence in patients with T1 NMIBC.^7^ The clinical benefit of each treatment has been well demonstrated. However, in clinical practice, two or more different types of therapies (e.g., second TUR and intravesical BCG therapy) are performed after the initial TURBT, and the outcomes in patients with NMIBC who received them have not been fully clarified.

Clinical practice guidelines for NMIBC recommend stratification of patients with NMIBC into low‐, intermediate‐, or high‐risk groups and provide risk‐adapted optimal surveillance and treatment strategies to minimalize the probability of disease recurrence and progression.^4^, ^8^ However, in real‐world clinical practice, a large percentage of patients are not optimally managed according to guidelines, and poor adherence to guideline recommendations may be correlated with unfavorable outcomes.^9^ Therefore, the present study aimed to investigate the level of adherence to guideline‐recommended therapies in patients with NMIBC and analyze the outcomes of those who did and did not undergo them.

## METHODS

### Ethics statements

This retrospective study was approved by the Institutional Review Board of Tohoku University Graduate School of Medicine (approval number: 2018‐1‐450), and it conforms to the provisions of the Declaration of Helsinki. Informed consent was obtained through an online opt‐out process.

### Study design and population

The records of individual patients who underwent TURBT between January 2009 and December 2016 at nine institutions participating in the Tohoku Urological Evidence‐based Medicine Study Group and Kyoto University Hospital were analyzed. The inclusion criteria were as follows: histologically confirmed NMIBC, minimum follow‐up of 3 years when patients did not experience disease recurrence or progression, and a minimum follow‐up period of 3 months if patients experienced recurrence or progression. The exclusion criteria were as follows: patients with a history of MIBC, non‐urothelial carcinoma, and NMIBC who underwent definitive treatment (radiotherapy or radical cystectomy). This study included patients who developed upper urinary tract urothelial carcinoma, underwent radical nephroureterectomy, and then developed intravesical recurrence; however, the European Association of Urology (EAU) risk‐stratification system does not include patients with newly diagnosed NMIBC with upper urinary tract recurrence. Thus, we excluded these patients.

### Data collection

Study variables at the initial TURBT included age, sex, tumor stage, grade, multiplicity (single or multiple), largest tumor diameter (<3 or ≥3 cm), 2019 EAU risk group, presence of carcinoma in situ (CIS), and tumor status (primary or recurrent). The clinicopathological characteristics of patients with NMIBC who received intravesical BCG therapy or a second TUR were investigated. Information on BCG therapy (the dose, total number of instillations, and whether the initially predetermined dosage and duration of treatment were completed), second TUR (the period between the initial TURBT and second TUR, and pathological findings at the second TUR), and IPIC (the type of chemotherapeutic agents administered intravesically immediately after the initial TURBT) was also evaluated. Recurrence was defined as intravesical recurrence after the initial TURBT. Progression was defined as the development of muscle‐invasive disease (T2 or higher) and metastasis to lymph nodes or other distant organs. Patients with NMIBC were stratified into low‐, intermediate‐, and high‐risk groups according to the 2019 EAU risk‐stratification system.^10^ To evaluate the clinical benefit of each therapy, we investigated recurrence‐free survival (RFS) in individual patients with intermediate‐ and high‐risk tumors who underwent a second TUR, BCG therapy, and IPIC. RFS was calculated as the period from the initial TURBT to the first recurrence, and patients who developed progression after the initial TURBT were excluded when RFS was evaluated. To assess the level of compliance with the guideline, the proportions of patients with each risk group of NMIBC who underwent the aforementioned therapies recommended in the 2019 EAU guideline were calculated; additionally, those at academic and non‐academic facilities were compared. Patients with T1 NMIBC were identified as complying with the guideline only when they received both maintenance BCG therapy and a second TUR (compliance group); those who underwent only one of both therapies and induction‐only BCG (including both induction‐only BCG and a second TUR) were classified as not complying with the guideline (non‐compliance group). Patients with intermediate‐risk tumors were considered to adhere to the guideline only when they received either IPIC followed by maintenance BCG therapy or IPIC followed by additional adjuvant intravesical chemotherapy (compliance group); and those who received only IPIC, induction‐only BCG, maintenance BCG (without IPIC), additional adjuvant intravesical chemotherapy (without IPIC), or none of the aforementioned therapies were classified as not complying with the guideline (non‐compliance group). This study compared RFS between compliance and non‐compliance groups by using propensity score‐matched analysis. When patients received additional BCG therapy following induction BCG, they were considered to be treated with “maintenance BCG therapy” even if the duration of treatment or dose failed to meet the standards recommended by the 2019 EAU guideline.^10^

### Statistical analysis

Continuous variables were expressed as median and interquartile range (IQR), and categorical variables were presented as absolute numbers and percentages. The Kaplan–Meier method with the log‐rank test was used to estimate and compare RFS between the groups. Categorical variables were compared using the Pearson chi‐squared test. Clinicopathological variables including age, sex, tumor status (primary or recurrent), tumor multiplicity, and largest tumor diameter were used for propensity score matching in patients with intermediate‐risk NMIBC; and age, sex, tumor status, stage, presence of CIS, grade, tumor multiplicity, and largest tumor diameter were used for that in patients with T1 NMIBC. Patients were matched at a 1:1 ratio. Differences were considered statistically significant at *p* < 0.05. When multiple comparisons between three and four groups were performed, the Bonferroni method was used, with *p*‐values <0.017 and 0.008 being considered statistically significant, respectively. All statistical analyses were performed using EZR (version 1.61; Saitama Medical Center, Jichi Medical University, Saitama, Japan), which is a graphical user interface for R.^11^

## RESULTS

In total, 1204 patients from 10 institutions were included in this study. The median age of the entire cohort at the initial TURBT was 71 years (IQR, 64–79 years). Table 1 shows the clinicopathological characteristics of patients treated and not treated with intravesical BCG therapy or a second TUR. Among patients with T1 NMIBC (*n* = 330), 184 (55.8%) did not undergo a second TUR. Among the patients with high‐risk tumors, 478 (74.0%) were not treated with maintenance BCG therapy. Among patients with intermediate‐risk tumors, 41 (9.0%) and 23 (5.1%) received maintenance BCG therapy or additional adjuvant intravesical chemotherapy, respectively. Information on BCG therapy, second TUR, and IPIC is shown in Table 2. Of the patients who underwent BCG therapy, approximately 70% were treated with a full dose of BCG and completed the initially intended dosage and duration of BCG therapy. Table 3 displays the compliance rates in the whole cohort and those in academic or non‐academic facilities. Except for IPIC in patients with low‐ and intermediate‐risk tumors, academic facilities exhibited significantly higher compliance rates than non‐academic facilities.

**TABLE 1:** Clinicopathological characteristics of patients who did and did not undergo intravesical BCG therapy or a second TUR.

Variable and number	Number (%)				
	BCG			Second TUR	
	No BCG	Induction‐only	Maintenance	No second TUR	Second TUR
Age, years					
<70 (*n* = 536)	366 (68.3%)	71 (13.2%)	99 (18.5%)	453 (84.5%)	83 (15.5%)
≥70 (*n* = 668)	452 (67.7%)	107 (16.0%)	109 (16.3%)	590 (88.3%)	78 (11.7%)
Sex					
Male (*n* = 972)	650 (66.9%)	139 (14.3%)	183 (18.8%)	842 (86.6%)	130 (13.4%)
Female (*n* = 232)	167 (72.0%)	39 (16.8%)	26 (11.2%)	201 (86.6%)	31 (13.4%)
Tumor stage					
Ta (*n* = 831)	650 (78.2%)	78 (9.4%)	103 (12.4%)	818 (98.4%)	13 (1.6%)
Tis (*n* = 41)	5 (12.2%)	17 (41.5%)	19 (46.3%)	40 (97.6%)	1 (2.4%)
T1 (*n* = 330)	160 (48.5%)	83 (25.2%)	87 (26.1%)	184 (55.8%)	146 (44.2%)
Tumor grade (WHO 2004/2016)					
Low (*n* = 474)	408 (86.1%)	27 (5.7%)	39 (8.2%)	470 (99.2%)	4 (0.8%)
High (*n* = 492)	251 (51.0%)	100 (20.3%)	141 (28.7%)	366 (74.4%)	126 (25.6%)
Tumor grade (WHO 1973)					
G1 (*n* = 139)	123 (88.5%)	9 (6.5%)	7 (5.0%)	138 (99.3%)	1 (0.7%)
G2 (*n* = 566)	428 (75.6%)	59 (10.4%)	79 (14.0%)	530 (93.6%)	36 (6.4%)
G3 (*n* = 280)	119 (42.5%)	86 (30.7%)	75 (26.8%)	186 (66.4%)	94 (33.6%)
Tumor multiplicity					
Solitary (*n* = 543)	452 (83.2%)	40 (7.4%)	51 (9.4%)	486 (89.5%)	57 (10.5%)
Multiple (*n* = 621)	356 (57.3%)	125 (20.1%)	140 (22.5%)	522 (84.1%)	99 (15.9%)
Largest tumor diameter, cm					
<3 (*n* = 937)	680 (72.6%)	125 (13.3%)	132 (14.1%)	842 (89.9%)	95 (10.1%)
≥3 (*n* = 197)	125 (63.5%)	33 (16.8%)	39 (19.8%)	147 (74.6%)	50 (25.4%)
Presence of CIS					
No (*n* = 1038)	792 (76.3%)	112 (10.8%)	134 (12.9%)	913 (88.0%)	125 (12.0%)
Yes (*n* = 162)	22 (13.6%)	65 (40.1%)	75 (46.3%)	126 (77.8%)	36 (22.2%)
2019 EAU risk groups					
Low (*n* = 103)	103 (100%)	0 (0%)	0 (0%)	103 (100%)	0 (0%)
Intermediate (*n* = 455)	382 (84.0%)	32 (7.0%)	41 (9.0%)	451 (99.1%)	4 (0.9%)
High (*n* = 646)	332 (51.4%)	146 (22.6%)	168 (26.0%)	489 (75.7%)	157 (24.3%)
Tumor status					
Primary (*n* = 964)	678 (70.4%)	128 (13.3%)	158 (16.3%)	813 (84.3%)	151 (15.7%)
Recurrent (*n* = 240)	139 (57.7%)	50 (20.7%)	51 (21.6%)	230 (95.8%)	10 (4.2%)

Abbreviations: BCG, bacillus Calmette–Guérin; CIS, carcinoma in situ; EAU, European Association of Urology; TUR, transurethral resection; WHO, World Health Organization.

**TABLE 2:** BCG treatment schedule and pathological results of the second TUR and intravesically administered chemotherapeutic agents immediately after the initial TURBT.

Variable	Number (%)
BCG (*n* = 386)	
Number of BCG instillations	
1–8 (induction only)	178 (46.1%)
7–15 (maintenance)	161 (41.7%)
16–21	40 (10.4%)
≥22	6 (1.6%)
Unknown	1 (0.3%)
BCG dose	
Full	279 (72.3%)
One‐half	88 (22.8%)
One‐third	19 (4.9%)
Planned BCG instillation completed	
Yes	267 (69.2%)
No	119 (30.8%)
Second TUR (*n* = 161)	
Duration between the initial and second TUR (weeks, median, interquartile range)	6 (5–7)
Residual tumor	
No residual tumors	90 (55.9%)
Ta (without CIS)	25 (15.5%)
CIS (with or without Ta tumors)	25 (15.5%)
T1 (with or without CIS)	17 (10.6%)
Stage unknown	4 (2.5%)
Immediate postoperative instillation of chemotherapy (*n* = 924)	
Chemotherapeutic agent	
Mitomycin C	400 (43.2%)
Epirubicin or Pirarubicin	522 (56.5%)
Other agents	2 (0.2%)

Abbreviations: BCG, bacillus Calmette–Guérin; CIS, carcinoma in situ; TUR, transurethral resection; TURBT, transurethral resection of bladder tumor.

**TABLE 3:** Proportion of patients with each risk group of NMIBC treated with adjuvant intravesical therapy and a second TUR.

	All (*n* = 1204)	Academic hospitals (*n* = 520)	Non‐academic hospitals (*n* = 684)	*P*‐value
IPIC in patients with low‐ and intermediate‐risk NMIBC	458/557 (82.2%)	190/240 (79.2%)	268/317 (84.5%)	0.081
Administration of maintenance BCG therapy in patients with high‐risk NMIBC	168/646 (26.0%)	109/279 (39.1%)	59/367 (16.1%)	<0.001
Administration of maintenance BCG therapy in patients with T1 NMIBC	87/330 (26.4%)	47/120 (39.2%)	40/210 (19.0%)	<0.001
Performance of a second TUR in patients with T1 NMIBC	146/330 (44.2%)	106/120 (88.3%)	40/210 (19.0%)	<0.001
Administration of maintenance BCG therapy and performance of a second TUR in patients with T1 NMIBC	55/330 (16.7%)	45/120 (37.5%)	10/210 (4.8%)	<0.001
Administration of maintenance BCG therapy or additional adjuvant intravesical chemotherapy in patients with intermediate‐risk NMIBC	64/455 (14.1%)	49/219 (22.4%)	15/236 (6.4%)	<0.001

Abbreviations: BCG, bacillus Calmette–Guérin; IPIC, immediate postoperative instillation of chemotherapy; NMIBC, non‐muscle‐invasive bladder cancer; TUR, transurethral resection.

**FIGURE 1:**
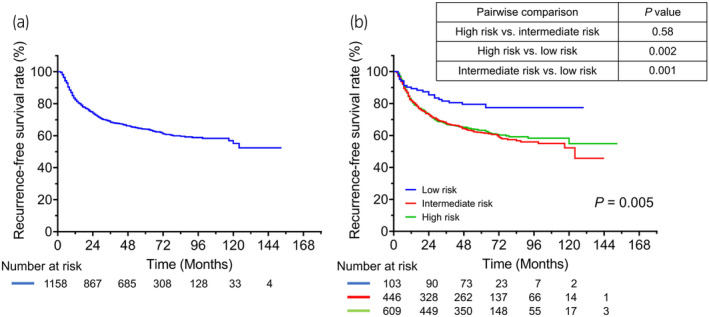
Kaplan–Meier plots displaying RFS. Panel (a) represents RFS in the entire cohort. Panel (b) represents RFS stratified according to the low‐, intermediate‐, and high‐risk groups classified by the 2019 EAU risk‐stratification system. EAU, European Association of Urology; RFS, recurrence‐free survival.

**FIGURE 2:**
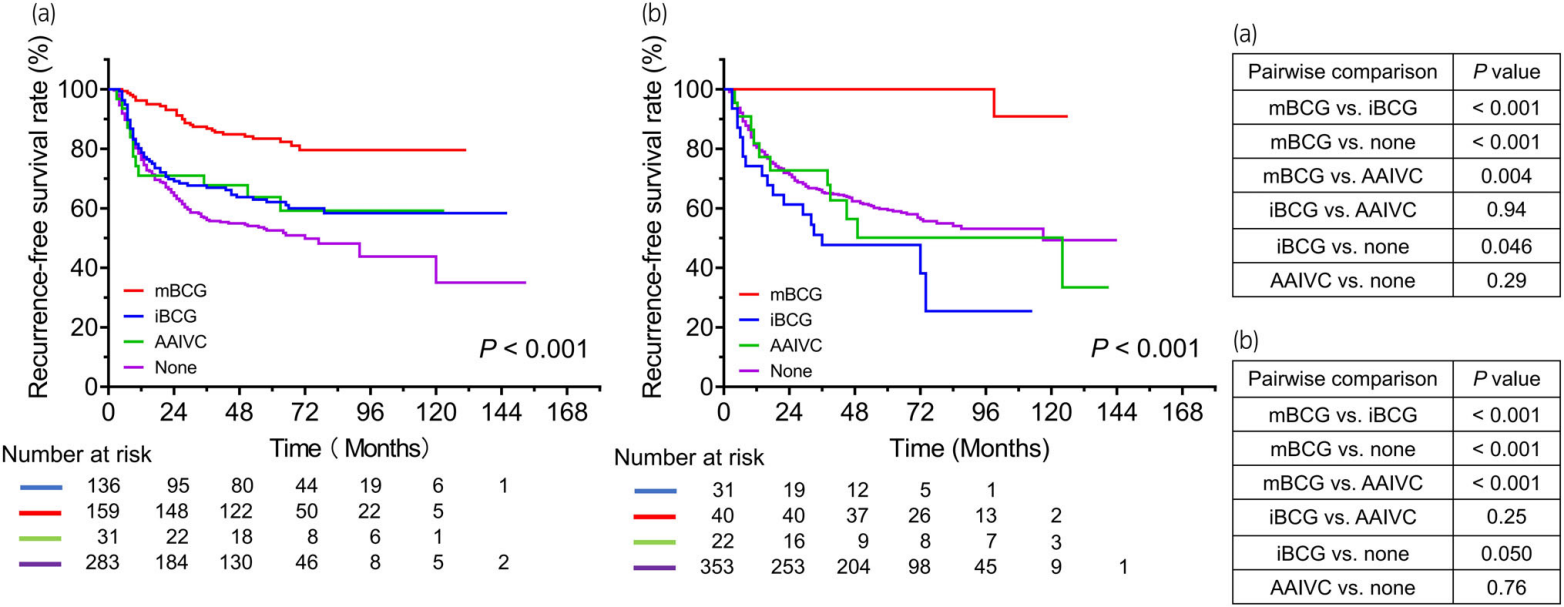
Kaplan–Meier curves for RFS in patients with (a) high‐ and (b) intermediate‐risk non‐muscle‐invasive bladder cancer. RFS is stratified according to adjuvant intravesical therapy. AAIVC, additional adjuvant intravesical chemotherapy; BCG, bacillus Calmette–Guérin; iBCG, induction‐only bacillus Calmette–Guérin; mBCG, maintenance bacillus Calmette–Guérin; RFS, recurrence‐free survival.

**FIGURE 3:**
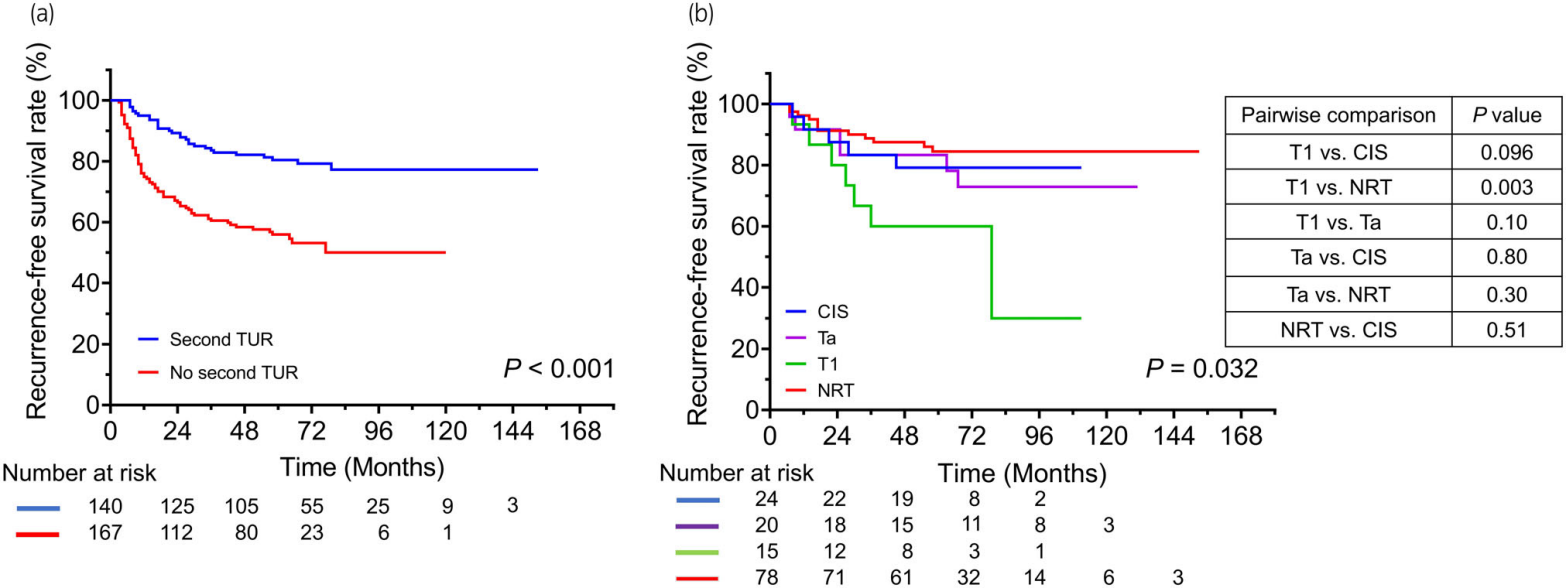
Kaplan–Meier plots of RFS in patients with stage T1 non‐muscle‐invasive bladder cancer at initial TURBT. Panel (a) represents RFS stratified according to the second TUR (second TUR vs. initial TURBT alone). Panel (b) represents RFS stratified according to the pathological findings at the second TUR. CIS, carcinoma in situ; NRT, no residual tumors; RFS, recurrence‐free survival; TUR, transurethral resection; TURBT, transurethral resection of bladder tumor.

**FIGURE 4:**
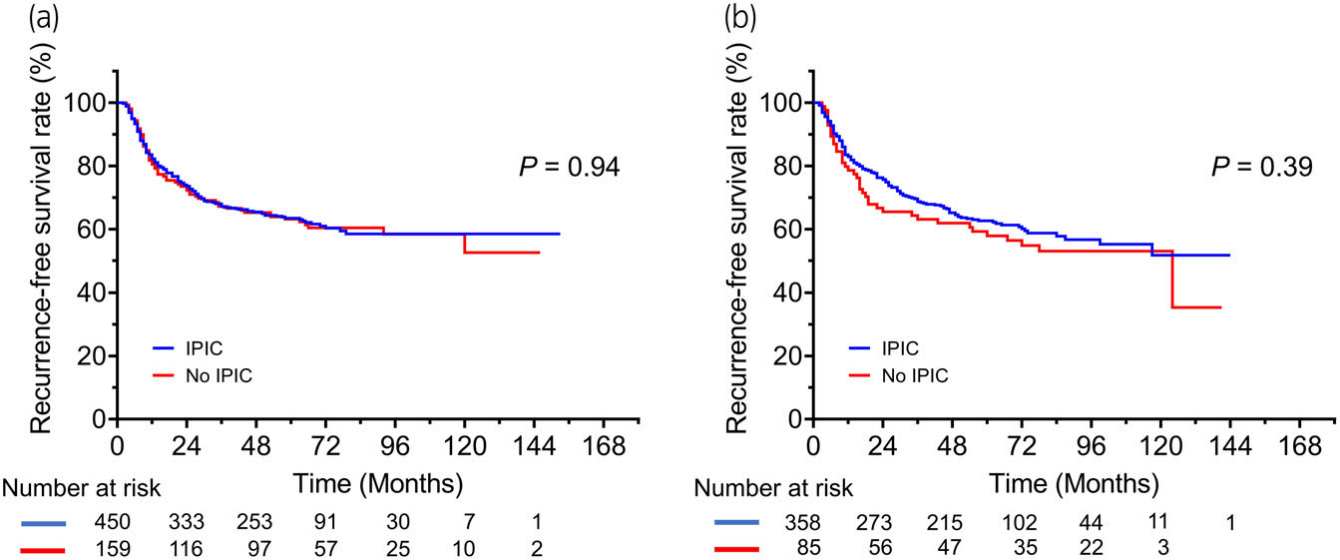
Kaplan–Meier curves for recurrence‐free survival in patients with (a) high‐ and (b) intermediate‐risk non‐muscle‐invasive bladder cancer stratified according to treatment (IPIC vs. no IPIC). IPIC, immediate postoperative instillation of chemotherapy.

**FIGURE 5:**
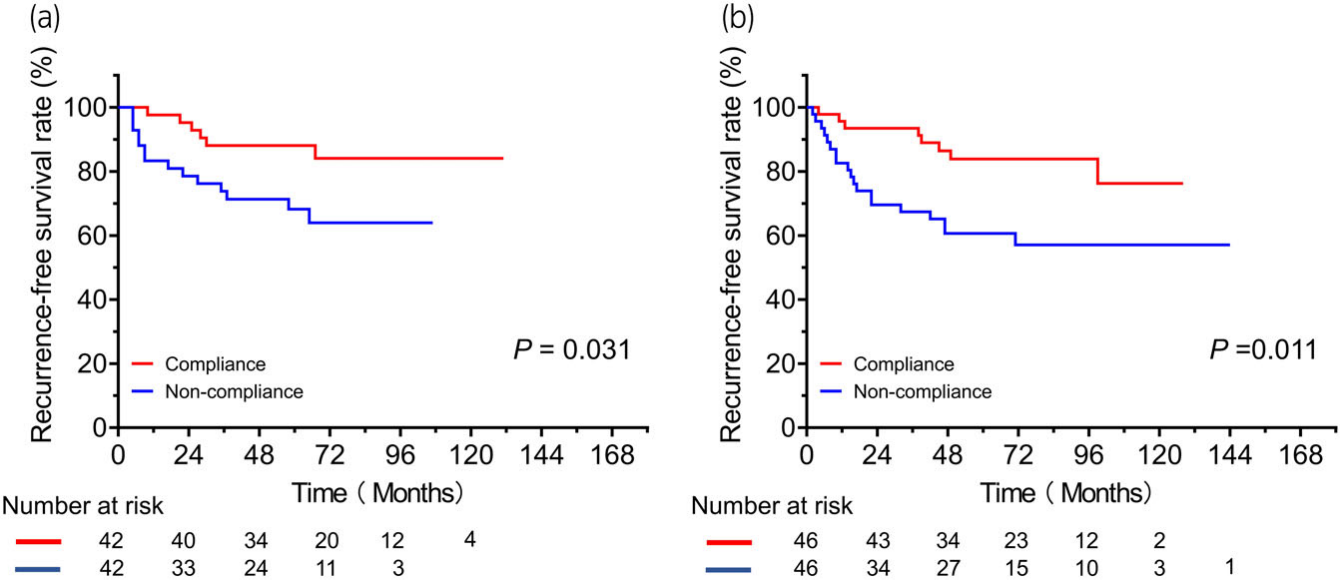
(a) Kaplan–Meier plots displaying RFS in patients with stage T1 NMIBC who received both maintenance BCG therapy and a second TUR (compliance group), and propensity‐matched patients who did not receive them (non‐compliance group). (b) Kaplan–Meier curve for RFS in patients with intermediate‐risk NMIBC who underwent either IPIC followed by maintenance BCG therapy or IPIC followed by additional adjuvant intravesical chemotherapy (compliance group), and propensity‐matched patients who received only IPIC, induction‐only BCG, maintenance BCG (without IPIC), additional adjuvant intravesical chemotherapy (without IPIC), or none of the aforementioned therapies (non‐compliance group). BCG, bacillus Calmette–Guérin; IPIC, immediate postoperative instillation of chemotherapy; NMIBC, non‐muscle‐invasive bladder cancer; RFS, recurrence‐free survival; TUR, transurethral resection.

Over a median follow‐up duration of 56 months, 429 (37.0%) patients experienced disease recurrence after the initial TURBT. Figure 1a,b shows RFS in the entire cohort and patients with low‐, intermediate‐, and high‐risk tumors. The 3‐year RFS rate in the entire cohort was 68.4%. Significant differences in RFS were found between the low‐ and intermediate‐risk groups (*p* = 0.001) and between the low‐ and high‐risk groups (*p* = 0.002). Figure 2a,b shows RFS in high‐ and intermediate‐risk patients who did and did not receive adjuvant intravesical therapy, including induction‐only BCG therapy, maintenance BCG therapy, or additional adjuvant intravesical chemotherapy, respectively. Patients with high‐ and intermediate‐risk tumors who received maintenance BCG therapy had significantly better RFS than those treated with other therapies. Figure 3 shows RFS for patients with T1 NMIBC who did and did not undergo a second TUR. Significantly better RFS was found in patients with T1 NMIBC who underwent a second TUR than in those who did not (*p* < 0.001) (Figure 3a). Patients who were diagnosed with T1 NMIBC at the initial TURBT and received a second TUR were divided into four groups as follows: patients without residual tumors at the second TUR; patients with Ta tumors (without CIS) at the second TUR; patients with T1 tumors (with or without CIS) at the second TUR; and patients with CIS (with or without Ta tumors) at the second TUR. Significant differences in RFS were found only between patients with T1 tumors at the second TUR and those without residual tumors (*p* = 0.003; Figure 3b). Figure 4 shows RFS in intermediate‐ and high‐risk patients treated with IPIC. There was no significant difference in RFS in the high‐risk group between treatments (Figure 4a, *p* = 0.94). Additionally, the difference in RFS in patients with intermediate‐risk tumors between treatments was not significant (Figure 4b, *p* = 0.39). Figure 5a shows RFS in patients with T1 tumors who did or did not receive a second TUR and maintenance BCG therapy. Patients with T1 NMIBC who received them (compliance group) showed significantly better RFS than propensity‐matched patients who did not (non‐compliance group) (*p* = 0.031). As shown in Figure 5b, patients with intermediate‐risk tumors who received either IPIC followed by maintenance BCG therapy or IPIC followed by additional adjuvant intravesical chemotherapy (compliance group) exhibited significantly greater RFS than propensity‐matched patients who did not receive either of the aforementioned therapies (non‐compliance group) (*p* = 0.011).

## DISCUSSION

Various mechanisms, including incomplete resection, tumor re‐implantation, and the field change cancerization effect, have been considered responsible for the disease recurrence of NMIBC.^12^ There are evidence‐based, comprehensive strategies to counteract those mechanisms; however, the recurrence rate remains high, as reported previously.^6^, ^7^, ^10^, ^12^ In a previous study, very few patients with NMIBC were managed in a manner recommended by the guidelines, and a significant improvement in outcome was found in patients with NMIBC who received at least half of the recommended care.^9^ The result suggested that poor adherence to guideline recommendations was partially responsible for recurrence.

Intravesical BCG therapy is superior to TURBT alone and TURBT with additional adjuvant intravesical chemotherapy in preventing disease recurrence, as reported previously.^4^ In a recent systematic review and meta‐analysis of RCTs, a 21% reduction in risk ratios of recurrence was observed in patients with NMIBC treated with maintenance BCG therapy compared with those who underwent induction‐only BCG therapy.^13^ Guidelines from EAU and the Japanese Urological Association (JUA) state that patients with intermediate‐ and high‐risk tumors should be treated with maintenance BCG therapy; however, a BCG shortage has become a global concern, and several guidelines allow adaptation of the dose or duration of BCG therapy based on its availability.^10^, ^14^, ^15^, ^16^ Herein, patients with intermediate‐ and high‐risk NMIBC treated with maintenance BCG therapy demonstrated significantly better RFS than those who underwent induction‐only BCG therapy (Figure 2a,b).

A systematic review concluded that a second TUR might improve the outcome of patients diagnosed with T1 NMIBC.^7^ Herein, 55.8% of patients diagnosed with T1 NMIBC at the initial TURBT did not undergo a second TUR, and significantly poor RFS was found in patients with T1 NMIBC who did not receive a second TUR compared with those who did (Figure 3a). The current study's results indicate that a second TUR has the potential to improve the outcomes in patients with T1 NMIBC. Patients with T1 NMIBC at the initial TURBT with T1 histology at the second TUR may be at high risk of disease progression and are possible candidates for radical cystectomy; our data showed that the RFS in these patients was significantly worse than that in patients with T1 NMIBC at the initial TURBT without residual tumors at the second TUR (Figure 3b).^17^ Pathological findings at the second TUR may have a significant impact on the prognosis of patients diagnosed with T1 NMIBC after the initial TURBT; however, the guidelines do not provide treatment strategies for these patients based on pathological findings at the second TUR.^4^, ^18^

Maintenance BCG therapy and second TUR are recommended for patients with T1 NMIBC in the guidelines.^10^, ^15^ In some previous studies, better outcomes were observed in high‐risk patients treated with a second TUR followed by BCG therapy than in those treated with BCG therapy alone.^19^, ^20^ In the current propensity score‐matched analysis, patients with T1 NMIBC treated with a second TUR, followed by maintenance BCG therapy, showed significantly superior RFS compared with those not treated with both therapies, suggesting that adherence to guideline‐recommended therapies improves patients' outcome.

In a recent systematic review and individual patient data meta‐analysis of RCTs, IPIC demonstrated a 14% reduction in the 5‐year recurrence rate; however, IPIC cannot provide clinical benefit for patients with a prior recurrence rate of >1 recurrence/year, and the European Organization for Research and Treatment of Cancer (EORTC) recurrence score ≥5.^6^ Consequently, the 2019 EAU guideline did not recommend IPIC for these patients.^10^ The current data available for analysis lack information on EORTC recurrence score and the prior recurrence rate per year, and it is possible that intermediate‐risk patients with a prior recurrence rate of >1 recurrence/year or an EORTC recurrence score ≥5 are identified as complying with the guideline. IPIC may not bring clinical benefit to patients with high‐risk tumors, and accordingly, the guidelines do not strongly recommend IPIC.^10^, ^21^ In the current study, 73.1% of high‐risk patients received IPIC, and that did not produce a significant reduction in the risk of recurrence. A systematic review reported that 53.0% of presumed low‐ and intermediate‐risk patients underwent IPIC.^22^ Herein, 80.8% of intermediate‐risk patients underwent IPIC. However, there was no significant difference in RFS between the treatments (Figure 4b), and it is unclear why IPIC did not show treatment efficacy.

A wide gap between routine practice for patients with NMIBC and guideline recommendations has been advocated in previous reports.^9^, ^23^, ^24^, ^25^ Multiple factors, including patient‐related, condition‐related, therapy‐related, cancer‐related, and socioeconomic factors, are associated with non‐compliance to guidelines.^22^ However, identifying the primary causes of not providing guideline‐recommended therapies is difficult, and detailed approaches to improve guideline adherence have not been shown.^25^ As an attempt to reduce an evidence‐practice gap, theory‐informed behavior change interventions have been applied; awareness of the evidence‐practice gap is the first important step to achieving behavioral change necessary to improve guideline adherence.^26^ Herein, a higher level of guideline adherence was generally observed in academic facilities compared with non‐academic facilities (Table 3). To our knowledge, in Japan, a large‐scale research study on guideline adherence for NMIBC has not been performed.^22^ An investigation with a sufficient number of cases to fully grasp the current situation (guideline adherence), followed by an assessment of potential barriers to the implementation of guideline recommendations and the development of methods to overcome these barriers, may lead to improvement in guideline adherence.^26^

In the present study, 46 patients (3.8% of the entire cohort) experienced progression after the initial TURBT. The current study did not include patients treated with definitive therapies after the initial TURBT; additionally, it lacked data on those who experienced disease recurrence after the initial TURBT followed by the development of progression. Therefore, our data could not be used to precisely estimate the patients who developed progression. It is important to investigate the association between guideline‐recommended therapies and disease progression. A further study is needed to clarify that. In our study, there was no significant difference in RFS between patients with intermediate‐ and high‐risk tumors. Tumor multiplicity (multiple), larger maximum tumor diameter (≥3 cm), and high prior recurrence rate (>1 recurrence per year), which were factors common to both risk groups, were more strongly associated with disease recurrence than the tumor grade (high), presence of CIS, and T category (T1 or Tis); this might explain the lack of difference in RFS between the high‐ and intermediate‐risk groups.^27^

This study has some limitations. The primary limitation is its retrospective design, and the patients analyzed herein did not receive a standardized follow‐up regimen. Moreover, the correlation between RFS and BCG dose or duration of maintenance therapy has not yet been investigated. During the research period, the JUA and EAU guidelines were updated several times, and the risk‐stratification system and recommended treatment strategy for each risk group were changed; and maintenance BCG therapy was recommended to patients with high‐risk tumors by the 2015 and 2019 JUA guidelines but not by the 2009 JUA guideline explicitly.^4^, ^10^, ^16^, ^28^, ^29^, ^30^ Therefore, patients with NMIBC who complied with the JUA or EAU guidelines at the time of treatment might be classified as not complying with the 2019 EAU guideline. Thus, such cases were considered as non‐compliance with the 2019 EAU guideline in this study. We performed a propensity score‐matched analysis aimed at comparing RFS between patients with T1 tumors who did and did not receive therapies recommended by the 2019 EAU guideline; however, a similar analysis for patients with high‐risk tumors was not carried out. We believe that, regardless of these limitations, our data provide real‐world outcomes of patients treated with a second TUR, intravesical therapy, and their combinations and information on the level of adherence to guideline recommendations.

In conclusion, our data revealed that compliance with guideline recommendations was poor, and unfavorable outcomes were observed in patients with NMIBC who did not receive guideline‐recommended therapies. The results of the current study suggest that improvements in adherence to guidelines may contribute to improved clinical outcomes.

## AUTHOR CONTRIBUTIONS

Takuma Sato: Conceptualization; Data curation; Formal analysis; Writing—original draft; Writing—review & editing. Takeshi Sano: Data curation; Writing—review & editing. Sadafumi Kawamura: Data curation; Writing—review & editing; Resources. Yoshihiro Ikeda: Writing—review & editing; Resources. Kazuhiko Orikasa: Data curation; Writing—review & editing; Resources. Takaki Tanaka: Data curation; Writing—review & editing; Resources. Atsushi Kyan: Writing—review & editing; Resources. Shozo Ota: Writing—review & editing; Resources. Satoru Tokuyama: Writing—review & editing; Resources. Hideo Saito: Writing—review & editing; Resources. Koji Mitsuzuka: Writing—review & editing. Shinichi Yamashita: Writing—review & editing. Yoichi Arai: Conceptualization; Writing—review & editing. Takashi Kobayashi: Writing—review & editing; Resources. Akihiro Ito: Supervision; Writing—review & editing.

## CONFLICT OF INTEREST STATEMENT

The authors declare no conflict of interest.

## APPROVAL OF THE RESEARCH PROTOCOL BY AN INSTITUTIONAL REVIEWER BOARD

The protocol for this research project has been approved by the Ethics Committee at Tohoku University Graduate School of Medicine (approval number: 2018‐1‐450), and it conforms to the provisions of the Declaration of Helsinki.

## INFORMED CONSENT

Informed consent was obtained in the form of online opt‐out.

## REGISTRY AND REGISTRATION NO. OF THE STUDY/TRIAL

N/A.

## ANIMAL STUDIES

N/A.

## Data Availability

The data supporting the findings of this study are available from the corresponding author upon reasonable request.

## References

[1] van Rhijn BW , Burger M , Lotan Y , Solsona E , Stief CG , Sylvester RJ , et al. Recurrence and progression of disease in non‐muscle‐invasive bladder cancer: from epidemiology to treatment strategy. Eur Urol. 2009;56:430–442. 19576682 10.1016/j.eururo.2009.06.028

[2] Cox E , Saramago P , Kelly J , Porta N , Hall E , Tan WS , et al. Effects of bladder cancer on UK healthcare costs and patient health‐related quality of life: evidence from the BOXIT trial. Clin Genitourin Cancer. 2020;18:e418–e442. 32144049 10.1016/j.clgc.2019.12.004 PMC7427321

[3] van den Bosch S , Alfred WJ . Long‐term cancer‐specific survival in patients with high‐risk, non‐muscle‐invasive bladder cancer and tumour progression: a systematic review. Eur Urol. 2011;60:493–500. 21664041 10.1016/j.eururo.2011.05.045

[4] Babjuk M , Burger M , Capoun O , Cohen D , Compérat EM , Dominguez Escrig JL , et al. European Association of Urology guidelines on non‐muscle‐invasive bladder cancer (Ta, T1, and carcinoma in situ). Eur Urol. 2022;81:75–94. 34511303 10.1016/j.eururo.2021.08.010

[5] Oddens J , Brausi M , Sylvester R , Bono A , van de Beek C , van Andel G , et al. Final results of an EORTC‐GU cancers group randomized study of maintenance bacillus Calmette‐Guérin in intermediate‐ and high‐risk Ta, T1 papillary carcinoma of the urinary bladder: one‐third dose versus full dose and 1 year versus 3 years of maintenance. Eur Urol. 2013;63:462–472. 23141049 10.1016/j.eururo.2012.10.039

[6] Sylvester RJ , Oosterlinck W , Holmang S , Sydes MR , Birtle A , Gudjonsson S , et al. Systematic review and individual patient data meta‐analysis of randomized trials comparing a single immediate instillation of chemotherapy after transurethral resection with transurethral resection alone in patients with stage pTa‐pT1 urothelial carcinoma of the bladder: which patients benefit from the instillation? Eur Urol. 2016;69:231–244. 26091833 10.1016/j.eururo.2015.05.050

[7] Cumberbatch MGK , Foerster B , Catto JWF , Kamat AM , Kassouf W , Jubber I , et al. Repeat transurethral resection in non‐muscle‐invasive bladder cancer: a systematic review. Eur Urol. 2018;73:925–933. 29523366 10.1016/j.eururo.2018.02.014

[8] Matsumoto H , Shiraishi K , Azuma H , Inoue K , Uemura H , Eto M , et al. Clinical practice guidelines for bladder cancer 2019 update by the Japanese Urological Association: summary of the revision. Int J Urol. 2020;27:702–709. 32564429 10.1111/iju.14281

[9] Chamie K , Saigal CS , Lai J , Hanley JM , Setodji CM , Konety BR , et al. Quality of care in patients with bladder cancer: a case report? Cancer. 2012;118:1412–1421. 21823107 10.1002/cncr.26402 PMC3213323

[10] Babjuk M , Burger M , Compérat EM , Gontero P , Mostafid AH , Palou J , et al. European Association of Urology guidelines on non‐muscle‐invasive bladder cancer (TaT1 and carcinoma in situ)—2019 update. Eur Urol. 2019;76:639–657. 31443960 10.1016/j.eururo.2019.08.016

[11] Kanda Y . Investigation of the freely available easy‐to‐use software ‘EZR’ for medical statistics. Bone Marrow Transplant. 2013;48:452–458. 23208313 10.1038/bmt.2012.244 PMC3590441

[12] Teoh JY , Kamat AM , Black PC , Grivas P , Shariat SF , Babjuk M . Recurrence mechanisms of non‐muscle‐invasive bladder cancer—a clinical perspective. Nat Rev Urol. 2022;19:280–294. 35361927 10.1038/s41585-022-00578-1

[13] Chen S , Zhang N , Shao J , Wang X . Maintenance versus non‐maintenance intravesical bacillus Calmette‐Guerin instillation for non‐muscle invasive bladder cancer: a systematic review and meta‐analysis of randomized clinical trials. Int J Surg. 2018;52:248–257. 29499363 10.1016/j.ijsu.2018.02.045

[14] Guallar‐Garrido S , Julián E . Bacillus Calmette‐Guérin (BCG) therapy for bladder cancer: an update. Immunotargets Ther. 2020;9:1–11. 32104666 10.2147/ITT.S202006 PMC7025668

[15] Japanese Urological Association , editor. Clinical practice guideline for bladder cancer 2019 edition. Tokyo: Igakutosho‐shuppan Ltd; 2019.

[16] Kubota Y , Nakaigawa N . Essential content of evidence‐based clinical practice guidelines for bladder cancer: the Japanese Urological Association 2015 update. Int J Urol. 2016;23:640–645. 27374472 10.1111/iju.13141

[17] Herr HW , Donat SM , Dalbagni G . Can restaging transurethral resection of T1 bladder cancer select patients for immediate cystectomy? J Urol. 2007;177:75–79. 17162005 10.1016/j.juro.2006.08.070

[18] Kitamura H , Kakehi Y . Treatment and management of high‐grade T1 bladder cancer: what should we do after second TUR? Jpn J Clin Oncol. 2015;45:315–322. 25583419 10.1093/jjco/hyu219

[19] Herr HW . Restaging transurethral resection of high risk superficial bladder cancer improves the initial response to bacillus Calmette‐Guerin therapy. J Urol. 2005;174:2134–2137. 16280743 10.1097/01.ju.0000181799.81119.fc

[20] Sfakianos JP , Kim PH , Hakimi AA , Herr HW . The effect of restaging transurethral resection on recurrence and progression rates in patients with nonmuscle invasive bladder cancer treated with intravesical bacillus Calmette‐Guérin. J Urol. 2014;191:341–345. 23973518 10.1016/j.juro.2013.08.022 PMC4157345

[21] Witjes JA . The final answer to the question of whether we should use a single postoperative instillation of chemotherapy after resection of pTa and pT1 bladder tumors. Eur Urol. 2016;69:245–246. 26251301 10.1016/j.eururo.2015.07.035

[22] Mori K , Miura N , Babjuk M , Karakiewicz PI , Mostafaei H , Laukhtina E , et al. Low compliance to guidelines in nonmuscle‐invasive bladder carcinoma: a systematic review. Urol Oncol. 2020;38:774–782. 32654948 10.1016/j.urolonc.2020.06.013

[23] Witjes JA , Palou J , Soloway M , Lamm D , Kamat AM , Brausi M , et al. Current clinical practice gaps in the treatment of intermediate‐ and high‐risk non‐muscle‐invasive bladder cancer (NMIBC) with emphasis on the use of bacillus Calmette‐Guérin (BCG): results of an international individual patient data survey (IPDS). BJU Int. 2013;112:742–750. 23452187 10.1111/bju.12012 PMC3933735

[24] Gontero P , Oderda M , Altieri V , Bartoletti R , Cai T , Colombo R , et al. Are referral centers for non‐muscle‐invasive bladder cancer compliant to EAU guidelines? A report from the vesical antiblastic therapy Italian study. Urol Int. 2011;86:19–24. 21196690 10.1159/000321926

[25] van Rhijn BW , Burger M . Bladder cancer: low adherence to guidelines in non‐muscle‐invasive disease. Nat Rev Urol. 2016;13:570–571. 27578042 10.1038/nrurol.2016.165

[26] French SD , Green SE , O'Connor DA , McKenzie JE , Francis JJ , Michie S , et al. Developing theory‐informed behaviour change interventions to implement evidence into practice: a systematic approach using the theoretical domains framework. Implement Sci. 2012;7:38. 22531013 10.1186/1748-5908-7-38 PMC3443064

[27] Sylvester RJ , van der Meijden AP , Oosterlinck W , Oosterlinck W , Witjes JA , Bouffioux C , et al. Predicting recurrence and progression in individual patients with stage Ta T1 bladder cancer using EORTC risk tables: a combined analysis of 2596 patients from seven EORTC trials. Eur Urol. 2006;49:466–477. 16442208 10.1016/j.eururo.2005.12.031

[28] Babjuk M , Oosterlinck W , Sylvester R , Kaasinen E , Böhle A , Palou‐Redorta J , et al. EAU guidelines on non‐muscle‐invasive urothelial carcinoma of the bladder, the 2011 update. Eur Urol. 2011;59:997–1008. 21458150 10.1016/j.eururo.2011.03.017

[29] Babjuk M , Böhle A , Burger M , Capoun O , Cohen D , Compérat EM , et al. EAU guidelines on non‐muscle‐invasive urothelial carcinoma of the bladder: update 2016. Eur Urol. 2017;71:447–461. 27324428 10.1016/j.eururo.2016.05.041

[30] Committee for Establishment of the Clinical Practice Guidelines for the Management of Bladder Cancer and the Japanese Urological Association . Evidence‐based clinical practice guidelines for bladder cancer (summary—JUA 2009 edition). Int J Urol. 2010;17:102–124. 20377834 10.1111/j.1442-2042.2010.02486.x

